# Diet and Energy-Sensing Inputs Affect TorC1-Mediated Axon Misrouting but Not TorC2-Directed Synapse Growth in a *Drosophila* Model of Tuberous Sclerosis

**DOI:** 10.1371/journal.pone.0030722

**Published:** 2012-02-03

**Authors:** Brian Dimitroff, Katie Howe, Adrienne Watson, Bridget Campion, Hyun-Gwan Lee, Na Zhao, Michael B. O'Connor, Thomas P. Neufeld, Scott B. Selleck

**Affiliations:** 1 Department of Genetics, Cell Biology and Development, Developmental Biology Center, University of Minnesota, Minneapolis, Minnesota, United States of America; 2 Graduate Program in Neuroscience, University of Minnesota, Minneapolis, Minnesota, United States of America; 3 Department of Biochemistry and Molecular Biology, The Pennsylvania State University, University Park, Pennsylvania, United States of America; National Institutes of Health (NIH), United States of America

## Abstract

The Target of Rapamycin (TOR) growth regulatory system is influenced by a number of different inputs, including growth factor signaling, nutrient availability, and cellular energy levels. While the effects of TOR on cell and organismal growth have been well characterized, this pathway also has profound effects on neural development and behavior. Hyperactivation of the TOR pathway by mutations in the upstream TOR inhibitors TSC1 (tuberous sclerosis complex 1) or TSC2 promotes benign tumors and neurological and behavioral deficits, a syndrome known as tuberous sclerosis (TS). In *Drosophila*, neuron-specific overexpression of Rheb, the direct downstream target inhibited by Tsc1/Tsc2, produced significant synapse overgrowth, axon misrouting, and phototaxis deficits. To understand how misregulation of Tor signaling affects neural and behavioral development, we examined the influence of growth factor, nutrient, and energy sensing inputs on these neurodevelopmental phenotypes. Neural expression of Pi3K, a principal mediator of growth factor inputs to Tor, caused synapse overgrowth similar to Rheb, but did not disrupt axon guidance or phototaxis. Dietary restriction rescued Rheb-mediated behavioral and axon guidance deficits, as did overexpression of AMPK, a component of the cellular energy sensing pathway, but neither was able to rescue synapse overgrowth. While axon guidance and behavioral phenotypes were affected by altering the function of a Tor complex 1 (TorC1) component, Raptor, or a TORC1 downstream element (S6k), synapse overgrowth was only suppressed by reducing the function of Tor complex 2 (TorC2) components (Rictor, Sin1). These findings demonstrate that different inputs to Tor signaling have distinct activities in nervous system development, and that Tor provides an important connection between nutrient-energy sensing systems and patterning of the nervous system.

## Introduction

The TOR signaling pathway is critical for a broad range of biological processes. TOR, a serine-threonine kinase, is best recognized for its role as a central regulator of cell and tissue growth [1], but recently it has been found to have other important activities independent of its effects on cell size and rates of cell division. TOR signaling influences development of the nervous system, controlling cell migration, synapse growth, and axon guidance [2], [3]. Disruption of the TOR pathway has profound consequences on neural development, as evidenced by the effects of abnormally elevated TOR signaling in humans with tuberous sclerosis [4]. Tuberous sclerosis (TS) is a dominant disorder produced by mutations affecting one of two proteins, TSC1 or TSC2. These proteins form a heteromeric complex that negatively regulates TOR activity, and mutations which impair the function of either component result in a high incidence of benign tumors, chronic seizures, and behavioral abnormalities such as attention deficit hyperactivity disorder (ADHD) and autism spectrum disorder (ASD) [5]. The physiological basis of these behavioral disorders is not known in detail, although studies in model organisms have shown that disruption of TOR signaling can affect synapse function, axon guidance, dendritic arborization, and cell migration during cortical assembly [6], [7], [8], [9].

In both humans and *Drosophila*, TOR associates with other proteins in the cell to form distinct TOR-containing complexes. TOR Complex 1 (TORC1) contains Raptor (Regulatory associated protein of mTOR), and is sensitive to the drug rapamycin. It is primarily involved in regulating protein translation through phosphorylation of the downstream targets S6-Kinase (S6K) and 4E Binding Protein (4EBP) [10]. TOR complex 2 (TORC2) contains Rictor (Rapamycin-insensitive companion of mTOR) and Sin1 (Stress-activated-protein-kinase-interacting protein 1). TORC2 is not affected by rapamycin, a TORC1-inhibitor and immunosuppressant, and in addition to having a role in actin dynamics [11], functions as the long-searched-for “PDK2”, capable of phosphorylating AKT at a key regulatory site. AKT is a serine-threonine kinase [12], [13] that not only inhibits TSC2 activity directly, but also influences apoptosis, metabolism, cell proliferation, and growth [14], [15]. Although much is known about the upstream inputs that control TORC1 activity, very little is known about regulation of TORC2 [16]. We are interested in examining the relative contribution of these two distinct complexes to the neurodevelopmental events governed by TOR, and in testing whether inputs that are important for regulating TOR-mediated growth will also play a role in regulating the effects of TOR on nervous system development and function.

As a key regulator of growth, TOR activity is controlled by a variety of inputs from nutrient and energy sensing systems, as well as a number of different growth factors [10]. Nutrient sensing is largely mediated by amino acid availability, however until recently it remained a mystery exactly how amino acids were able to influence TOR pathway activity. Recent studies have shed light on this question by identifying a family of molecules known as Rag GTPases, which are able to modify TOR activity in response to amino acid levels [17], [18], [19]. They most likely achieve this by facilitating increased interaction between TOR and Rheb (Ras homolog enriched in brain), a small GTPase directly downstream of TSC1/2 and directly upstream of TOR [20]. Cellular energy in the form of ATP is sensed by AMPK (AMP-activated Protein Kinase) [21]. When ATP levels are low, AMPK reduces the activity of the TOR pathway by promoting the inhibitory functions of TSC1/2. Growth factors, such as insulin and IGF-1 (Insulin-like growth factor 1), regulate TOR activity through the class I Phosphatidylinositol 3-kinase (PI3K) [14], [22]. Activation of PI3K produces the charged lipid molecule PIP_3_ (phosphatidylinositol (3,4,5)-triphosphates), which in turn activates a signaling cascade of kinases that governs the function of the TSC1/2 complex. A principal step in this cascade is the phosphorylation of AKT by PDK1 (Protein-dependent kinase 1), which occurs at a conserved site on AKT separate from the site of TORC2-mediated phosphorylation. Discovering how these various nutrient sensing, energy sensing, and growth factor mediated inputs to TOR differ in their effects on neurodevelopment is a key step towards understanding the overall processes that direct neural patterning and function. This knowledge will also aid in identifying possible candidates for pharmacological intervention in treating diseases where TOR signaling is misregulated, such as tuberous sclerosis.

Because TOR activity is greatly affected by nutritional and energy sensing inputs, we have an avenue for manipulating the function of this pathway through strictly environmental influences, such as diet composition and caloric intake. This provides a unique opportunity for evaluating gene-environment interactions, a fundamental component of disease susceptibility [23]. The degree to which environmental dietary factors affect normal development is largely an open question. Instead, we have focused on the relationship between diet and neural development in the context of TOR misregulation, such as occurs in humans with tuberous sclerosis. The possibility that dietary restriction could be a treatment option for patients with TS is not unprecedented. Dietary regimes such as the ketogenic diet, where total calories are reduced and lipids become the primary source of caloric intake, are already effectively being used to treat seizures, including those exhibited by individuals with TS [24].

Our study seeks to understand the neurological and behavioral abnormalities produced by hyperactivation of the TOR pathway using *Drosophila* as a model system. Complete loss-of-function of either *Tsc1* or *gigas*, the *Drosophila Tsc2* homolog, is lethal [25], [26], so we have created a model of TS in the fruit fly using neuron-directed overexpression of Rheb, the GTP-binding protein that is the immediate upstream activator of Tor. This provides a more modest activation of the pathway [27] and more accurately reflects the partial loss-of-function found in patients with TS, where heterozygosity for either *TSC1* or *TSC2* produces the disease. In *Drosophila* we have identified three different neurodevelopmental and behavioral phenotypes that result from *Rheb* overexpression: photoreceptor axon guidance abnormalities in the brain, deficits in normal phototaxis behavior, and increased size and hyperactivity of synapses at the neuromuscular junction (NMJ). We examined the influence of various genetic and environmental inputs to these Tor-misregulation abnormalities and tested their abilities to rescue each of the different phenotypes. Rheb and Pi3K showed distinct and separable roles in neural development, with Rheb hyperactivity producing a broader spectrum of abnormalities. Changes in diet, both nutrient composition and caloric content, affected neural patterning and behavioral responses, as did genetic manipulations of the critical energy sensor AMPK. Synaptic phenotypes responded differently compared to axon guidance and behavioral phenotypes, and we show that this is due to a dependence on different downstream Tor complexes. Taken together, these findings demonstrate that Tor signaling provides a critical link between metabolic factors and neural development, and that environmental influences such as diet can effect neural patterning and function.

## Results

### The TSC-TOR signaling system

**Figure 1:**
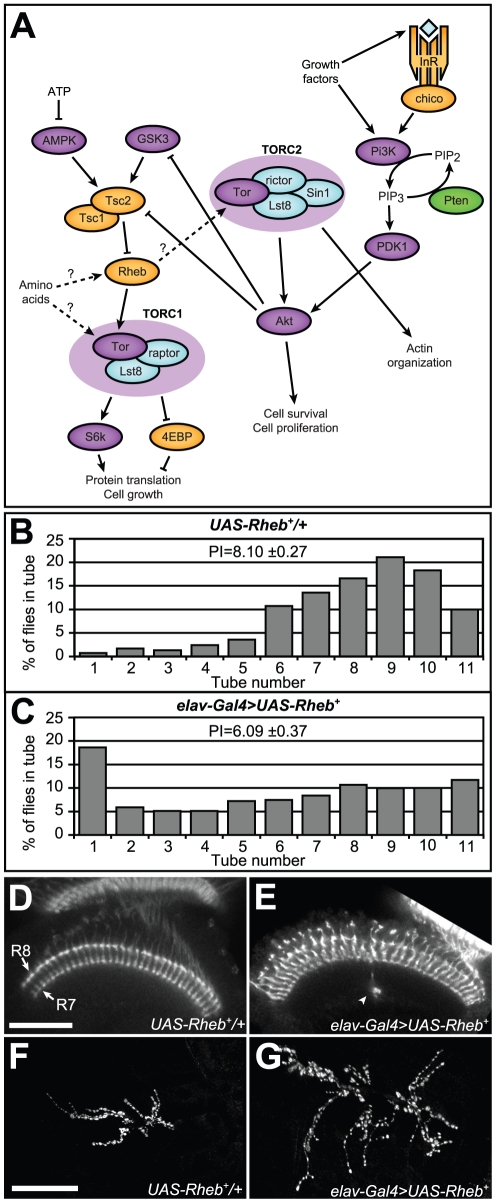
Rheb overexpresion causes defects in behavior, axon guidance, and synapse morphology. (A) Schematic diagram of the Tor pathway. Kinases are purple, Tor-complex components are blue, phosphatases are green, and other components are orange. The Tsc1/2 heterodimer inhibits Rheb, which in turn controls the activity of two Tor-containing complexes, TORC1 and TORC2. Relationships which are not fully-understood or could have multiple intermediary steps are shown as dashed arrows with question marks. (B, C) Phototaxis measurements in flies overexpressing Rheb in all neurons (*elav-Gal4>UAS-Rheb^+^*, n = 416) compared to control flies that have *UAS-Rheb* ^+^ transgenes but lacked the neuron-specific *Gal4* driver (*UAS-Rheb^+^/+*, n = 531). The percentage of flies in each tube of the phototaxis apparatus after 10 trials is shown, along with the phototaxis index (PI), a cumulative score where higher numbers indicate a stronger phototaxis response. (D, E) Optic lobes of pupal brains oriented with retinas toward the top of the photos. Visualization of photoreceptor projections with Anti-Chaoptin antibody staining reveals that R7 and R8 photoreceptor neurons form two highly-structured parallel rows of terminations in the medulla (arrows), with no axon bundles projecting beyond the R7 termination layer in controls. (E) *Rheb* overexpression in neurons caused some axon bundles to continue growing beyond their proper R7/R8 termination targets to eventually stop elsewhere within the medulla (arrowheads). (F, G) Anti-CSP (Cysteine string protein) staining at the larval NMJ reveals synaptic active zones known as boutons. Neuron-specific overexpression of *Rheb* resulted in neuromuscular synapses approximately 50% larger than corresponding synapses in animals lacking a *Gal4* driver. Scale bars are 50 microns.

The complex signaling system that includes Tsc1/Tsc2, Rheb, and Tor is outlined in Figure 1A [10]. Tor activity is controlled by a number of critical inputs, including growth factor signaling pathways mediated through Pi3K, metabolic energy regulators such as AMPK, and direct measures of amino acid availability. Nutrient-rich conditions that favor growth promote activation of Tor, while dietary or energy limitations reduce Tor activity [28]. In the cell, Tor associates with other proteins to form at least two unique Tor-containing complexes, TorC1 and TorC2, each with its own distinct binding partners and targets. TorC1, which contains Raptor, affects protein translation via S6k and 4EBP, while TorC2, which contains both Rictor and Sin1, influences actin organization and phosphorylates Akt, a critical regulator of several different cellular processes. The immediate upstream components most directly involved in regulating Tor activity are Rheb and Tsc1/Tsc2.

### A *Drosophila* model of TS

In *Drosophila*, overexpression of *Rheb* results in hyperactivation of the Tor pathway. The level of activation achieved in this manner is more modest than generating *Tsc1* or *gigas* knockouts, and more accurately reflects the levels of TOR activation seen in humans with TS, where heterozygosity for *TSC1* or *TSC2* mutations produces symptoms. Systemic overexpression of *Rheb* causes a high degree of lethality, so we used the *Gal4-UAS* system [29] to selectively express *Rheb* in neurons. This resulted in behavioral, morphological, and physiological abnormalities in the nervous system.

Positive phototaxis responses were used as a sensitive behavioral measure of nervous system function in this *Drosophila* model of TS. The phototaxis assay, developed by Seymour Benzer, provides a measure of responses to light in repeated trials of adult flies where 10 successive trials are conducted using a single apparatus [30]. Flies that show positive phototaxis for all ten trials end up in the 11^th^ tube of the phototaxis apparatus. The distribution of flies in the 11 tubes is therefore a measure of their phototaxis behavior (see Fig. 1B). The phototaxis index (PI) provides a consolidated measure of responses across an entire test group [31]. Neuronally-directed overexpression of *Rheb* produced phototaxis deficits and a significantly lower phototaxis index (PI = 6.1 in *Rheb* overexpressing flies, versus 8.1 in control flies lacking a *Gal4* driver, p<0.001 by Student's *t-*test).

Overexpression of *Rheb* in neurons had other neurodevelopmental consequences as well. Elevated signaling through the Tor pathway, mediated by either loss of *Tsc1* or increased levels of Rheb, resulted in axon guidance defects in the brain [27]. *Drosophila* have eight distinct photoreceptor neurons, R1–R8, and during development these project axons to specific termination sites in the brain [32]. By 40-hours after pupal formation (APF), the R7 and R8 photoreceptor axons terminate in a highly reproducible pattern in the medulla region. When viewed in optical cross-section, these terminations appear as regularly spaced rows of processes (Fig. 1D). Neuronal overexpression of *Rheb* resulted in a failure of some R7 and R8 photoreceptors to terminate at their proper locations (Fig. 1E, arrowheads). Sometimes these aberrant axon bundles looped back to terminate elsewhere in the medulla, while in some animals the axons simply stopped outside of the normal termination zone. The photoreceptor termination defects in transgenic flies overexpressing *Rheb* were relatively moderate compared to mosaic animals where a majority of retinal cells were homozygous mutant for *Tsc1* [27].

In addition to the photoreceptor axon guidance defects, we observed that hyperactivation of Tor signaling also greatly affected synapse development. Overexpression of *Rheb* in neurons caused substantial synaptic overgrowth at the neuromuscular junction (Fig. 1F, G), altering the balance between the number of synaptic boutons and the size of the underlying muscle, a tightly controlled relationship during normal growth and development [27], [33]. Animals bearing *elav-Gal4>UAS-Rheb^+^* transgenes sometimes showed more than a two-fold enlargement of NMJ synapses and were on average approximately 50% larger [27].

### Rheb activity is mediated through Tor

**Figure 2:**
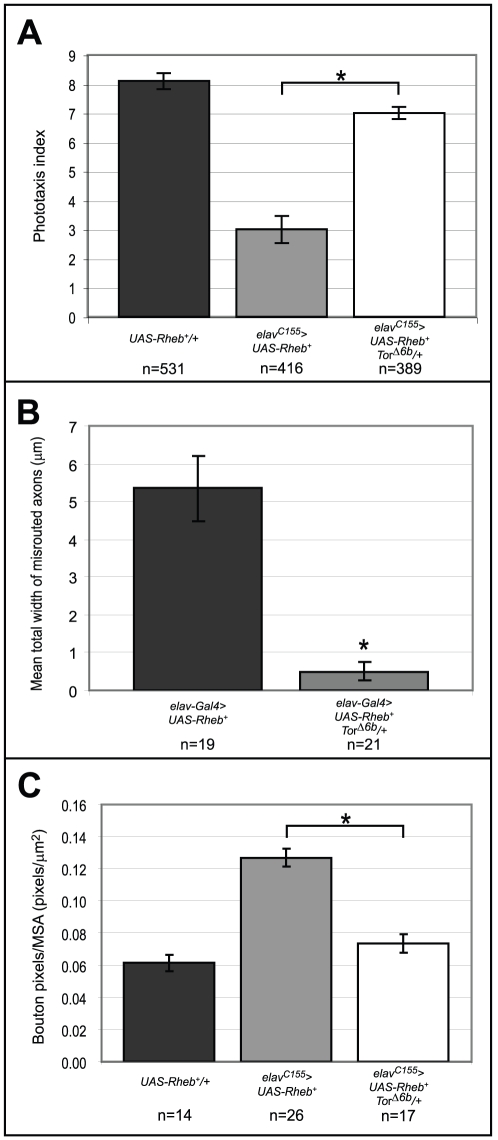
Rheb overexpression defects are Tor-dependent. (A–C) Heterozygosity for a *Tor* null mutation, *Tor^Δ6B^*, rescued Rheb-directed deficits in phototaxis behavior, axon guidance, and synapse expansion. (A) Neuronally-directed expression of *Rheb* (*elav^C155^>UAS-Rheb^+^*) caused a substantial drop in phototaxis response compared to controls (*UAS-Rheb^+^/+*). Heterozygosity for a *Tor* null allele (*elav^C155^>UAS-Rheb^+^ Tor^Δ6B^/+*) almost completely rescued this defect. (B) Measures of axon guidance misrouting showed a significant decrease in severity when *Tor* function was reduced by 50%. (C) Reduced *Tor* function rescued synapse overgrowth to nearly wild-type levels in animals with neuronally-directed (*elav-Gal4*) expression of *Rheb* (MSA = “Muscle Surface Area”). Asterisks denote p<0.05 using a two-tailed student's t-test. Note that different neuronal-directed *Gal4* drivers were used for assessing the influence of *Tor* function on Rheb-directed axon misrouting and synapse expansion. These two different *Gal4* drivers were selected for producing phenotypes at a penetrance and expressivity where *Tor-*dependence could readily be assessed.

Although Rheb is known to be directly upstream of Tor [34], we wanted to verify that these *Rheb* overexpression phenotypes were the result of increased Tor activation. Complete loss of Tor is lethal, so Tor function was reduced by 50% by creating animals heterozygous for a *Tor* null mutation. Reducing Tor function in the context of neuronal *Rheb* overexpression rescued phototaxis behavior almost to the level of controls (Fig. 2A). To assess the degree of photoreceptor axon misrouting, the widths of all axon bundles that failed to terminate at their proper locations were measured and averaged across the total number of optic lobes examined. The average width of misrouted axons in wild-type controls was essentially zero (data not shown). Heterozygosity for a *Tor* null mutation almost completely rescued Rheb-induced axon guidance abnormalities (Fig. 2B).

At the NMJ, overexpression of *Rheb* produced large synapses. The size of the synapse was measured by immunostaining with anti-Cysteine String Protein (CSP), a critical presynaptic component, and determining the number of pixels with staining above a background threshold. These “bouton pixel” counts were then divided by the surface areas of the underlying muscles, a method used for normalizing the size of the motoneuron process to the size of its target. This method of measuring synapse size is comparable to traditional bouton counts, but can be more quantitative when boutons are very small or indistinct, as is the case in animals with *Rheb* overexpression (see Methods). Reducing Tor function by 50% caused a significant rescue of the synapse overgrowth phenotype (Fig. 2C). Taken together with the phototaxis behavior and axon guidance results, it is evident that the range of neurodevelopmental deficits produced by *Rheb* overexpression all require Tor activity, demonstrating that this is a good model for studying the consequences of TOR signaling hyperactivation, as occurs in TS.

### Pi3K and Rheb have distinct activities in neural development

**Figure 3:**
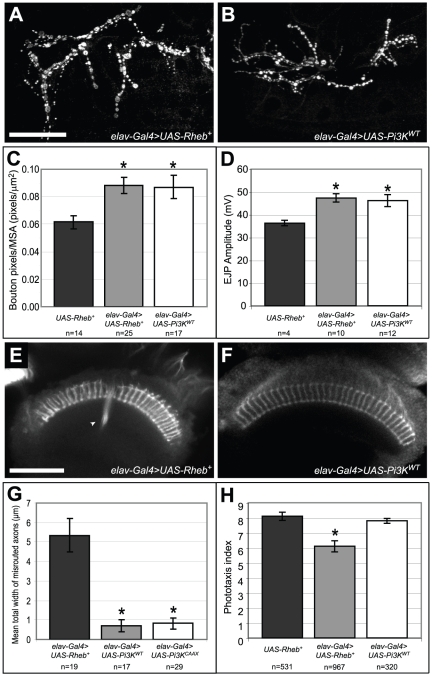
Pi3K and Rheb have similar effects on synapse size and function, but not on axon guidance or phototaxis behavior. (A–C) Neuronal expression of either *Pi3K* or *Rheb* had equivalent effects on synapse size, as measured by anti-CSP staining. (D) Suprathreshold electrical stimulation of the motoneuron produces synaptic transmission and depolarization of the underlying muscle known as an excitatory junctional potential (EJP) [33]. This physiological measurement provides a reproducible readout of synapse functionality. Compared to control animals lacking a neuron-specific *Gal4* driver, *Pi3K* expression and *Rheb* expression both caused similar increases in EJP amplitudes at the neuromuscular junction. (E–G) Anti-Chaoptin staining of photoreceptor axons revealed that, contrary to the axon guidance defects observed in animals overexpressing *Rheb* (arrowhead), axon guidance misroutings in *Pi3K*-expressing animals were extremely rare, even when we expressed the constitutively-activated transgene *UAS-Pi3K^CAAX^*. (H) Neuronal overexpression of *Pi3K* did not cause deficits in phototaxis behavior, unlike what was observed when flies overexpressed *Rheb*. Asterisks denote p<0.05 compared to control *UAS-Rheb^+^/+* animals using a two-tailed student's t-test statistic. Scale bars are 50 microns.

Pi3K and Rheb constitute two different upstream regulators of Tor, and their relative contributions to distinct neurobehavioral phenotypes were examined. At the larval NMJ, Pi3K and Rheb have been shown to have largely equivalent effects in promoting both NMJ expansion and increases in physiological responses to motoneuron stimulation [27]. While Rheb is immediately upstream of Tor, Pi3K conveys signaling information from growth factor receptors at the cell surface via multiple intermediates [14], [22]. The similar effects of Rheb and Pi3K expression at the NMJ prompted an analysis of the influence these two critical Tor signaling components have on other neurodevelopmental phenotypes, namely phototaxis and photoreceptor axon guidance. Consistent with previous reports, expressing *Rheb* or *PI3K* with an *elav-Gal4* transgene specific to neurons produced similar levels of synaptic expansion (Fig. 3A–C) and comparable increases in EJP amplitudes with either combination (Fig. 3D). However, using these same *Gal4-UAS* transgenes, *Rheb* and *Pi3K* overexpression produced very different effects on photoreceptor axon guidance and phototaxis. Whereas overexpression of *Rheb* caused substantial axon termination errors, *Pi3K* had virtually no effect on axon guidance in the visual system, even when expressed from a constitutively active transgene *Pi3K^CAAX^* [35](Fig. 3E–G). Similarly, *Pi3K* overexpression had no effect on phototaxis behavior, in contrast to the deficits observed when *Rheb* was overexpressed (Fig. 3H). These results demonstrate that despite having similar effects on the synapse, Rheb and Pi3k have distinct roles within the full spectrum of Tor-mediated neurodevelopmental events.

### Dietary rescue of behavioral deficits

**Figure 4:**
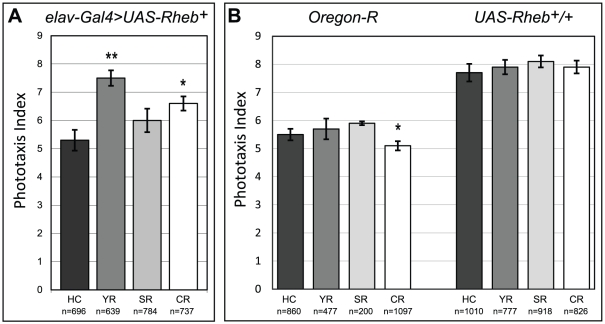
Changes in diet can rescue phototaxis behavior. (A) Yeast-restricted (YR) and calorie restricted (CR) diets significantly rescued phototaxis behavioral deficits in *Rheb*-overexpressing flies, compared to identical flies raised on a diet of rich, high-calorie (HC) food. Flies raised on a diet restricted only for sugar (SR) showed a slight trend toward improvement, but the results were not significant. (B) *Oregon-R*, a wild-type strain of *Drosophila* which phototaxes poorly but has normal levels of Tor pathway activity, did not show any improvement of phototaxis responses due to dietary changes. A small, statistically significant decrease in phototaxis performance was seen as a result of the calorie-restricted (CR) diet. Dietary changes did not affect phototaxis behavior in control flies which lacked a *Gal4* driver, and therefore did not overexpress *Rheb*. The number of animals (n) in each group is indicated. In all graphs, one asterisk denotes p<0.05 using a two-tailed student's t-test (compared against the HC diet) and two asterisks denotes p<0.001.

TOR was originally identified as a key regulator of cell and tissue growth in response to nutrition [1]. Conditions of plentiful nutrient and calorie availability are known to activate the TOR pathway and promote growth, whereas conditions of low food availability inhibit TOR and restrict growth. Nutrient availability is also a potentially important environmental modulator of neural development. As the behavioral, morphological, and physiological defects in our *Drosophila* model of TS are dependent on elevated Tor signaling, we examined the capacity of dietary restriction to modulate these effects. Flies were reared on four different diets: (1) a rich, high-calorie diet (high-calorie or HC, 1203 kcal/L), (2) a diet low in yeast, the primary source of lipids and amino acids, which also provides 28% fewer calories as a result (yeast-restricted or YR, 861 kcal/L), (3) a diet isocaloric to the yeast-restricted diet, but reduced instead for sugar, the primary source of carbohydrates (sugar-restricted or SR, 863 kcal/L), and (4) a diet restricted for both yeast and sugar, providing a low caloric content (calorie-restricted or CR, 521 kcal/L) [36]. Both the yeast-restricted (YR) and calorie-restricted (CR) diets significantly rescued phototaxis deficits in *Rheb*-overexpressing flies, with the YR food restoring this behavior to nearly wild-type levels (Fig. 4A). These changes in behavior were not a consequence of the dietary changes alone, since these diets had no effect in the absence of the *Gal4* transgene necessary for producing overexpression of *Rheb* (Fig. 4B). Control experiments with animals bearing *elav-Gal4* and a GFP-tagged transgene (*UAS-mCD8-GFP*) showed that these diets did not affect *Gal4-*directed expression levels measured at either the NMJ or in the third instar larval CNS (data not shown). We also examined the possibility that diet did not show an effect on wild-type fly behavior since these animals had such a robust phototaxis response that any enhancements from dietary changes were obscured. To test this possibility we assessed the effects of dietary change in a fly stock with a much lower baseline phototaxis score. Early studies in *Drosophila* established that different wild-type strains display varying degrees of positive phototaxis [37], and one strain with a particularly low level of phototaxis is *Oregon-R* (*Ore-R*). We raised *Ore-R* flies on each of the four diets and found no evidence of enhanced phototaxis behavior (Fig. 4B). In these tests a small, yet significant, decrease in performance was observed with the calorie-restricted diet, but there was no evidence of any improvement of behavior such as we observed in animals overexpressing *Rheb*. Therefore, the improvement in phototaxis performance observed in *elav-Gal4>UAS-Rheb^+^* flies is mediated by caloric restriction and represents a specific gene and environment interaction (*Rheb* overexpression and diet).

### Dietary rescue of Tor-mediated axon guidance abnormalities

**Figure 5:**
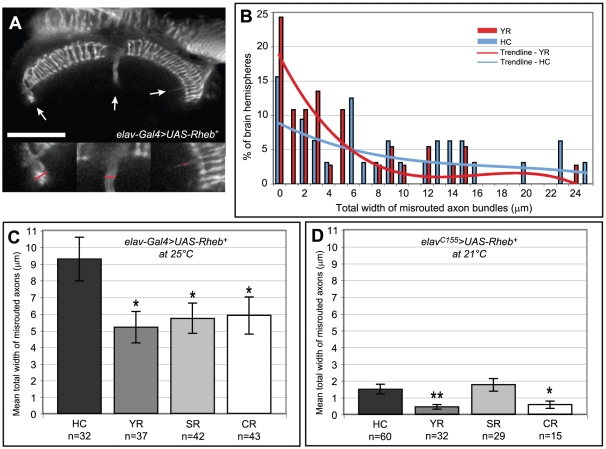
Dietary changes can rescue axon misrouting defects in photoreceptor neurons. (A) In pupal brains with neuronally-directed overexpression of *Rheb* (*elav-Gal4>UAS-Rheb^+^*), anti-Chaoptin staining revealed photoreceptor axon bundles that failed to stop at their proper targets and continued to grow into other areas of the brain (arrows). The inset images at the bottom of this panel (2× magnified) contain red bars that illustrate our technique for measuring across the width of these misrouted axon bundles. By adding these measurements together we achieved a semi-quantitative measure for the severity of axon guidance defects within each individual brain hemisphere. (B) Dietary restriction in *Rheb*-overexpressing animals shifted the distribution of brains with axon guidance problems away from the more severe defects and towards more mild phenotypes (compare YR and HC diets). This was measured by the percentage of brain hemispheres (penetrance) with wider bundles of axon misroutings versus the percentage of brains with smaller misroutings or no misroutings at all. (C) Averaging the width of misrouted axon bundles across all the brains in a particular group provides an alternative measure of axon misrouting severity. The restricted diets significantly rescued axon guidance defects in *Rheb*-overexpressing animals compared to the same flies raised on rich, high-calorie (HC) food. (D) We greatly reduced the level of *Rheb* overexpression by using a different neuron-specific *Gal4* driver, *elav^C155^*, and rearing the flies at a temperature that limits Gal4 activity, 21°C. Under these conditions, axon misrouting defects were much milder and could only be rescued by the YR and CR diets, both of which are restricted in their levels of yeast, the primary source of lipids and amino acids. Reducing sugar alone (the SR diet) had no effect. In all graphs, one asterisk denotes p<0.05 using a two-tailed student's t-test (compared against the HC diet) and two asterisks denotes p<0.001. Scale bar is 50 microns.

Our results thus far demonstrate that a behavioral aspect of nervous system dysfunction produced by Tor hyperactivation can be corrected by dietary restriction, but what about a morphological deficit? *Rheb* overexpression in the nervous system produced a moderate defect in photoreceptor axon targeting, and the capacity of caloric restriction to ameliorate this developmental deficit was examined. Elevated levels of Tor signaling caused R7 and R8 photoreceptor axons to grow past their intended targets in the brain (Fig. 5A, arrows). To quantify the severity of this defect, the widths of all aberrant axon bundles that failed to terminate were measured (as indicated by the red bars in Fig. 5A, insets), and the total width of misrouted axons was determined for each brain. Compared to control animals raised on the high-calorie (HC) diet, *Drosophila* raised on the yeast-restricted (YR) diet displayed a shift away from the more severe defects and a corresponding increase in the proportion of milder defects (Fig. 5B). This rescue is more readily seen by comparing the mean total widths of all misrouted axons within each group (Fig. 5C). Interestingly, when *Rheb* was expressed at this moderately high level, all three diet-restricted foods showed a significant capacity to rescue the axon guidance phenotype.

The capacity of different diets to provide rescue of axon guidance was explored further using conditions where the degree of misrouting was less severe. It seemed possible that any differences between these diets might only be observed at modest levels of Tor activation, where smaller changes in Tor signaling would have a more detectable effect. To produce lower levels of Tor activation, flies were reared at a reduced temperature (21°C versus 25°C) where the *Gal4* transcriptional activator protein is less active and therefore directs a lower level of *Rheb* expression. This technique resulted in an approximately 5-fold reduction in the severity of axon guidance defects (compare HC diets in Fig. 5C to 5D). Within this context, the yeast-restricted and calorie-restricted diets still showed a significant capacity to rescue, but the sugar-restricted diet did not (Fig. 5D). Both YR and CR diets limit lipids and amino acids, whereas SR is specifically limiting for carbohydrates. Since the YR and SR diets contain equal amounts of calories, these results suggest that for this level of Tor pathway activation it is the composition of each diet that is the key determinant of rescue, rather than the overall caloric content.

Since the experiments described thus far restrict caloric input by altering the food content, it was important to determine if the rates of food consumption and absorption were equivalent across all diets. A radioactive label was seeded into the food, and third instar larvae were allowed to ingest the labeled food for a 6-hour interval. The levels of radioactivity incorporated into the larvae were then determined, which provides a measure of both food ingestion and nutrient absorption [38]. No significant differences in the rates of food uptake were observed for any of the four different diets (Fig. S1), thus the effects of diet on behavioral and morphological development were not confounded by differing levels of food consumption.

### Effects of diet on Tor-mediated synapse abnormalities

**Figure 6:**
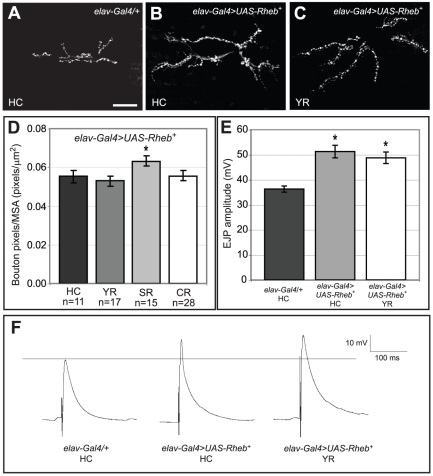
Dietary restriction does not rescue Rheb-mediated overgrowth or electrophysiological changes at neuromuscular synapses. (A, B) *Rheb* overexpression caused an increase in the CSP-expressing pixels (boutons) in larval motoneurons at the neuromuscular junction, as shown by anti-CSP staining. (C, D) Dietary restriction showed no significant rescue of the *Rheb*-overexpression phenotype at the NMJ, and restriction of sugar (SR) actually caused a modest increase in the CSP-stained regions of the boutons, corrected for muscle size (by dividing by the muscle-surface-area). (E) The hyperfunctional excitatory junctional potential (EJP) responses seen in *Rheb*-overexpressing animals could not be rescued by a dietary regime that was particularly effective at rescuing both phototaxis behavior and axon misrouting (compare *elav-Gal4>UAS-Rheb* ^+^ animals raised on the YR diet, n = 11, to *elav-Gal4>UAS-Rheb* ^+^ animals raised on the HC diet, n = 11. For *elav-Gal4/+* control animals raised on HC food, n = 7). (F) Representative traces of recorded EJP measurements clearly illustrate the elevated amplitudes observed when *Rheb* is overexpressed, regardless of diet. Asterisks denote p<0.05 using a two-tailed student's t-test compared against the HC diet in D, and against *elav-Gal4/+* control animals in E. Scale bar is 50 microns.

In addition to testing the effects of diet restriction on phototaxis deficits and axon patterning defects, the influence of dietary inputs on the synaptic abnormalities produced by *Rheb* overexpression was also examined. Hyperactivation of the Tor pathway resulted in substantial overgrowth of synapses at the neuromuscular junction (Fig. 6A, B), as visualized by staining for the presynaptic marker Cysteine-String Protein (CSP). Despite its ability to rescue other *Rheb*-overexpression phenotypes, diet restriction showed no capacity to rescue synaptic overgrowth (Fig. 6B–D). Animals raised on YR or CR diets showed the same degree of bouton-per-muscle-area expansion as flies raised on the high-calorie diet. Flies raised on the sugar-restricted diet actually showed an increase in synaptic overgrowth. Control experiments showed that reducing caloric content was also unable to limit synapse growth in animals without overexpression of *Rheb* in the nervous system (*elav-Gal4>UAS-mCD8-GFP*; data not shown).

Hyperactivation of Tor signaling also caused changes in the electrophysiological function of the NMJ. Suprathreshold stimulation of the motoneuron evokes a depolarization at the muscle known as the excitatory junctional potential (EJP) [33], [39](see Fig. 6F). Overexpression of Rheb caused an abnormal increase in the magnitude of this response, and raising flies on YR food was not able to rescue this defect (Fig. 6E, F). The inability of dietary restriction to affect either the NMJ growth or the physiological changes mediated by hyperactivation of Tor indicates that nutritional signals have a smaller impact on these Rheb-mediated outcomes.

### Effects of activated AMPK on Tor misregulation

**Figure 7:**
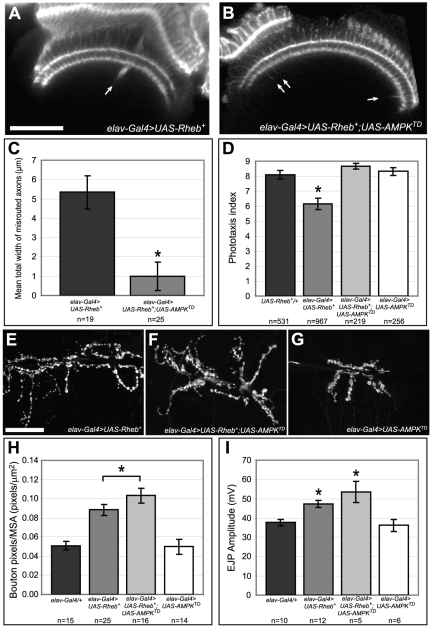
Constitutively active AMPK rescues Rheb-mediated axon guidance and phototaxis deficits, but not NMJ overgrowth or amplified EJP responses. (A–C) Expression of a constitutively activated *AMPK* transgene together with *Rheb^+^* greatly decreased the severity of the axon misrouting defects (arrows) normally seen in *Rheb*–overexpressing animals. (D) Co-expression of both *AMPK^TD^* and *Rheb^+^* in neurons also rescued the phototaxis deficits normally present in *Rheb*-expressing animals. When we expressed *AMPK^TD^* on its own, we did not see any change in phototaxis performance compared to controls or any measurable axon misrouting (data not shown). (E–H) At the larval NMJ, co-expression of a constitutively-activated *AMPK* in *Rheb*-overexpressing neurons did not rescue Rheb-mediated synaptic overgrowth, and in fact, caused an even greater increase in synapse size, despite the fact that *AMPK^TD^* had no effect on synapse growth when expressed on its own. (I) Similarly, co-expression of *AMPK^TD^* in *Rheb*-overexpressing neurons failed to rescue the elevated EJP amplitudes and, in fact, further exacerbated this defect. *AMPK* ^TD^ had no effect on synaptic response when expressed alone. Asterisks denote a two-tailed Student's t-test statistic of p<0.05. Scale bars are 50 microns.

Given the ability of dietary changes to alter the behavioral and axon guidance deficits of Tor hyperactivation, we examined whether genetically manipulating an energy-sensing input to Tor signaling, AMPK, could rescue the *Rheb* overexpression abnormalities we previously observed. In the cell, AMPK assess the ratio between the high-energy molecule ATP and its low-energy counterpart ADP [21]. Under low-energy conditions, AMPK positively regulates Tsc1/2 activity and causes a corresponding decrease in Tor pathway activity (see Fig. 1). Expressing a constitutively activated form of *AMPK*, *AMPK^TD^* [21], in neurons provided signals indicative of a low energy status. When *AMPK^TD^* and *Rheb^+^* were co-expressed in neurons there was a significant rescue of Rheb-mediated axon guidance and phototaxis abnormalities compared to animals with neuronal expression of *Rheb^+^* alone (Fig. 7 A–D). In previous experiments, dietary restriction was not able to rescue Tor-misregulation defects at the synapse, and a similar result was found for AMPK. Not only did *AMPK^TD^* expression fail to rescue the synaptic abnormalities (Fig. 7 E–I), it actually enhanced Rheb-mediated synapse overgrowth (Fig. 7H). Expression of *AMPK^TD^* alone, under the direction of *elav-Gal4*, did not affect NMJ size or EJP responses. It would appear, therefore, that the synaptic effects of AMPK are dependent on the baseline activity of Rheb and Tor.

### Identifying downstream components of Rheb-overexpression phenotypes

**Figure 8:**
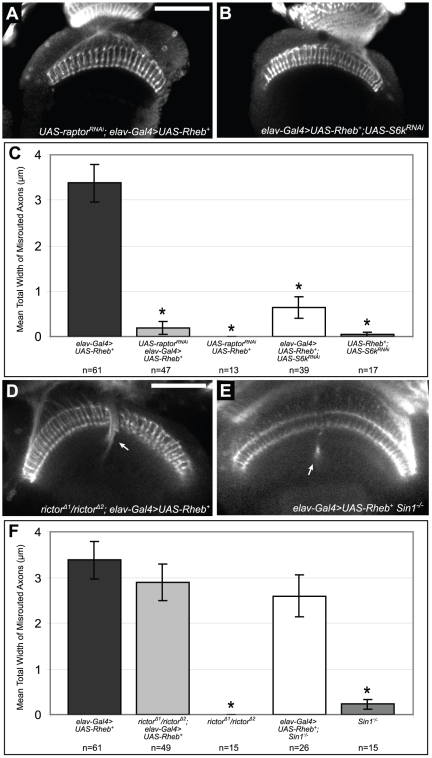
Rheb-mediated axon guidance defects are dependent on TorC1 downstream components, but not on TorC2. (A–C) *Rheb* was neuronally expressed in pupal brains along with *RNAi* constructs against either *raptor*, a principal component of Tor-complex 1, or *S6k*, an important downstream mediator of TorC1 activity. Genetic knockdown of either of these critical mediators of TorC1 signaling significantly rescued the axon misrouting defects normally observed in *Rheb*-overexpressing animals. (D–F) When *Rheb* was neuronally overexpressed in animals with null mutations in either of the critical TorC2 components *rictor* or *Sin1*, we saw no significant rescue of axon guidance defects (arrows). Although *Sin1* mutants did show a small degree of axon misrouting even in the absence of *Rheb* misexpression, this level of defect was not substantial enough to confound the interpretation of our primary results. Asterisks denote a two-tailed Student's t-test statistic of p<0.05 compared to *elav-Gal4>UAS-Rheb^+^* controls. Scale bars are 50 microns.

To determine how different inputs that influence Tor activity (calorie levels, AMPK, Rheb, Pi3K) affected some *Rheb*-overexpression phenotypes but not others, a series of experiments were undertaken to modulate Tor-containing complexes downstream of Rheb. Previous studies have established that nutrient levels, and in particular amino acids, regulate the growth control functions of TOR primarily by modulating the activity of TORC1, the Raptor-containing complex that affects translation, ribosome biogenesis, and autophagy [10], [20]. Because the axon guidance defects produced by *Rheb* overexpression were sensitive to changes in dietary composition, TorC1 was a logical signaling complex to test that could be essential for photoreceptor axon misrouting abnormalities. This hypothesis was examined by testing the ability of an *RNAi* construct targeted against *raptor*, a critical component of TorC1 [16], [40], to suppress the effects of neuronally-directed *Rheb* expression. Using the pan-neuronal driver *elav-Gal4*, *Rheb^+^* was co-expressed along with a *raptor* RNAi transgene. Knockdown of *raptor* resulted in almost complete rescue of Rheb-mediated axon guidance defects (Fig. 8A, C), suggesting that this is indeed a TorC1-dependent output of Tor hyperactivation. RNAi knockdown of *S6k*, a major downstream target of TorC1-signaling, also significantly reduced the axon misroutings produced by overexpression of *Rheb^+^* in neurons, supporting the conclusion that this phenotype is largely a TorC1-directed process (Fig. 8B, C). To rule out the possibility that the presence of two *UAS-*containing transgenes titrated the level of *Gal4* protein, and produced rescue by simple reduction of *Rheb* expression, we determined the level of synapse expansion mediated by *elav-Gal4>UAS-Rheb* when a second *UAS-mCD8-GFP* was present in the stock. In this case we observed a 3-fold expansion of the NMJ (data not shown), a level comparable to previously published findings from our group (2.2 fold) [41]. We have also conducted control experiments where we examined the expression levels of two tagged *UAS-*transgenes, either when only one (*UAS-mCD8-GFP*) or both (*UAS-mCD8-GFP* and *UAS-RFP*) were present in the animal bearing the *elav-Gal4* driver. Expression of *UAS-mCD8-GFP* at the NMJ was unaffected by the presence of the second *UAS-*transgene (data not shown), indicating that titration of *Gal4* protein was not a confounding factor in these experiments.

TorC2 is a regulator of actin dynamics, cell morphological changes, and dendritic arborization [9], [11]. Mutations compromising the function of *Sin1* and *rictor*, two unique TorC2 components, have been described and provide a means of selectively compromising this signaling entity. Null mutations in either of these genes are viable, allowing these alleles to be placed in the context of a fly with neuronally-directed *Rheb^+^* expression. Null mutations in either *rictor* or *Sin1* failed to show a statistically significant effect on Rheb-mediated axon guidance defects (Fig. 8D–E), indicating that TorC2 is not involved in this particular output of elevated Tor signaling.

**Figure 9:**
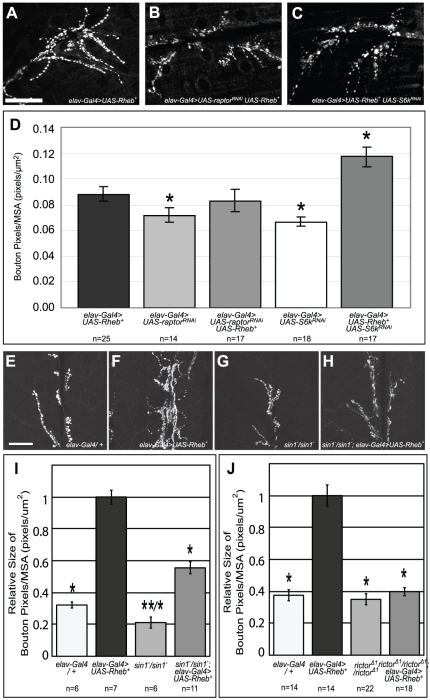
Rheb-mediated synapse overgrowth was not rescued by knockdown of TorC1 signaling, however it was rescued by loss of the TorC2 components *sin1* and *rictor*. (A) Anti-CSP staining of third-instar larval NMJs shows considerable overgrowth of motoneuron boutons in *Rheb*-overexpressing animals. (B–D) *RNAi* knockdown of either the TorC1 component *raptor* or the TorC1 downstream mediator *S6K* failed to decrease the severity of synapse overgrowth defects when *Rheb* was overexpressed. Reducing *S6K* function actually worsened the severity of this phenotype. (E–J) In contrast, homozygous loss of the TorC2 components *sin1* and *rictor* significantly rescued synaptic overgrowth in *Rheb*-overexpressing animals, indicating that this is a TorC2-dependent event. Asterisks denote a two-tailed Student's t-test statistic of p<0.05 compared to *elav-Gal4>UAS-Rheb^+^*(*) or to *elav-Gal4/+* (**) controls. Scale bars are 50 microns.

In contrast to axon guidance defects, the synapse overgrowth phenotypes of *Rheb* overexpression were not suppressed by diet restriction or AMPK activity. To further explore these findings, TorC1 activity was inhibited with the same neuronally-directed *raptor* and *S6k* RNAi constructs that were effective in suppressing axon guidance defects mediated by Rheb. Neither the *S6k* nor *raptor* RNAi transgene was able to rescue Rheb-mediated synaptic overgrowth (Fig. 9A–D). To evaluate the role of TorC2 in *Rheb-*directed synapse overgrowth, *sin1* and *rictor* mutants were crossed into *elav-Gal4>UAS Rheb^+^* flies. Both *sin1* and *rictor* mutations were able to suppress the overgrowth of the NMJ mediated by overexpression of *Rheb* (Fig. 9E–J). *sin1* mutants also showed a significantly smaller synapse compared to controls (Fig. 9I), suggesting that TORC2 does play a role in the NMJ growth during normal development.

## Discussion

The TOR signaling pathway, long known for influencing growth control and tumor formation, has more recently been identified as an important pathway in nervous system development, controlling such cellular events as cell migration, axon guidance, synaptic expansion, and dendritic arborization [3], [7], [9], [42], [43], [44]. The central role of TOR as an integrator of signals from different metabolic and growth factor inputs has been well established with regards to growth control, but how these inputs affect TOR-directed neural development had not been previously examined. The TOR signaling pathway is rather unique in serving a critical role in both the regulation of metabolism and the control of developmental patterning. The fact that dietary influences are able to affect the morphological and physiological processes controlled by this system emphasizes that energy availability is not simply a “stop” or “go” input to development. Rather, neural patterning and metabolism are linked via this critical growth regulatory pathway.

### Pi3K and Rheb have distinct activities in neural patterning

**Figure 10:**
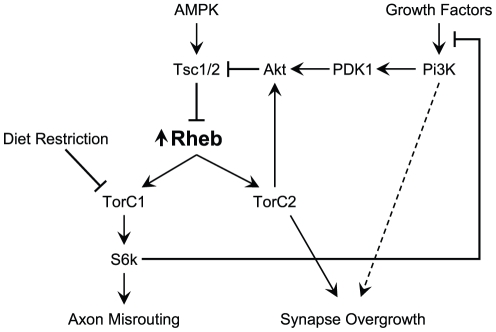
Axon guidance and synapse growth are controlled by separate branches of the Tor signaling pathway. A schematic diagram illustrating the effects of Tor-signaling elements on axon guidance and synapse growth. *Rheb*-mediated axon misrouting defects are dependent on signaling through TorC1 and S6k, and can be rescued by diet restriction or AMPK expression. Axon guidance is largely insensitive to changes in Pi3K. Synapse overgrowth mediated by *Rheb* overexpression is dependent on signaling through TorC2, is not rescued by caloric restriction or AMPK activation, and is exacerbated by knockdown of *S6k*, suggesting the removal of a feedback inhibition. Synapse overgrowth is also induced by *Pi3K* overexpression, similarly to *Rheb*.

We tested multiple upstream inputs to Tor signaling and found each one to have a varying ability to affect Tor-hyperactivation phenotypes (summarized in Fig. 10). For example, growth factor inputs through Pi3K produced the same level of synapse expansion as overexpression of *Rheb*, however Pi3K had no effect on axon misrouting or phototaxis behavior. This fits with earlier work from our group where we found that loss of *Pten*, an antagonist of Pi3K activity, in the *Drosophila* retina produced much milder photoreceptor misrouting defects compared to loss of *Tsc1*, the primary inhibitor of Rheb [27]. Rheb activates Tor directly, whereas Pi3K influences Tor through a series of molecular intermediaries, most notably Akt and Tsc1/Tsc2 [22]. Pi3K signaling certainly does play an important role in Tor pathway events such as responding to insulin signals and mediating inhibitory feedback mechanisms, yet Pi3K signaling also has a number of other downstream targets independent of Tor. It would seem that in photoreceptor axon guidance and phototaxis behavior Pi3K has only a modest impact on Tor signaling.

### Dietary and AMPK-mediated rescue of Rheb-induced axon guidance defects

Manipulation of metabolic inputs provided significant rescue of Tor-mediated axon guidance and phototaxis deficits. The most substantial rescue of *Rheb* overexpression phenotypes by diet came from the ones lowest in lipids and amino acids, namely YR and CR. This suggests caloric levels and content both affect Tor activity in the context of neural patterning. Indeed, different amino acids are known to have varying degrees of effect on TOR pathway activity. For example, restricting the level of either leucine or arginine alone results in nearly the same degree of TORC1 inactivation as restricting all amino acids [28]. These observations are relevant toward designing diets that are optimally suited for diminishing the effects of TOR hyperactivation. We note that more severe disruption of Tor signaling produced by complete loss of *Tsc1* function in the retina of a genetic mosaic animal was not rescued by dietary restriction (data not shown), indicating that an intact Tsc-Rheb-Tor axis is required for dietary effects. This is precisely the situation that exists in individuals with tuberous sclerosis since heterozygosity for *TSC1* or *TSC2* is causative for the disorder.

Expression of a constitutively activated AMPK in neurons also rescued Rheb-mediated misrouting of photoreceptor axons. This finding is consistent with current models where AMPK serves to activate the Tsc1/Tsc2 complex, thus limiting Tor activation under conditions of energy depletion. However, neither diet restriction nor AMPK activation rescued synapse overgrowth. In fact, expression of a constitutively activated AMPK enhanced the Rheb-mediated synapse overgrowth phenotype. This finding emphasizes that axon misrouting and synapse growth are fundamentally different Tor-regulated processes. If AMPK primarily affects reduction of TorC1 activity, perhaps the downregulation of TorC1 influences a feedback loop that enhances other Tor-dependent events at the NMJ. In fact, we have evidence that Rheb-directed synapse overgrowth is largely a TorC2-mediated process and is relatively insensitive to TorC1 activity.

A similar rescue of some Tor-hyperactivation phenotypes but not others was recently reported for a mouse model of TS in which *Tsc1* function had been eliminated in most neurons [7]. In this system, rapamycin treatment rescued abnormal cell body growth, myelination deficits, and neurofilament overenlargement, yet rapamycin had no effect on neuronal dysplasia and it only slightly rescued defects in dendritic spine density. These results illustrate a categorical difference between different outputs of TOR hyperactivation, and they demonstrate that various inputs to TOR signaling are specifically targeted to some functions of TOR and not others.

### TorC1 and TorC2 control distinct processes in neural development

Rapamycin treatment and amino acid restriction act primarily on TorC1 rather than TorC2. The ability of diet and AMPK activity to affect axon misrouting and phototaxis deficits in Rheb-overexpressing animals suggested that these defects were largely TorC1-dependent events. This model was supported by our finding that Rheb-directed axon misrouting was rescued by knockdown of the TorC1 component *raptor* or the primary downstream component of TorC1 signaling, *S6k*. These results are on the face of it in contrast with previously published findings that reductions in *S6K* function or rapamycin treatment (a TORC1-inhibitor) were unable to ameliorate axon guidance abnormalities in genetic mosaics where photoreceptor neurons are homozygous mutant for *Tsc1* [41]. It is important to point out the substantial difference in phenotype severity produced by loss of *Tsc1* in the retina versus overexpression of Rheb described here, a much milder phenotype. We interpret these findings that modulation of TORC1 function (rapamycin) and output (S6K) is only effective in altering the severity of the axon guidance abnormality in the context of an intact Tsc-Rheb-Tor axis. It is also possible that TORC1 is only one TOR-containing signaling complex that affects axon guidance, and in the presence of very high levels of TOR activity, TORC1-modulation cannot suppress the most severe phenotoypes. Axon guidance abnormalities produced by overxpression of Rheb were however, entirely insensitive to mutations in *rictor*, an essential TorC2-component. These findings show that disruptions of Tor pathway function that affect axon misrouting can be affected by TorC1-directed processes (Fig. 10) and we were unable to detect TORC2-modulation or regulation of axon routing processes in the visual system.

Unlike axon misrouting defects, synapse overgrowth was not rescued by dietary restriction or knockdown of TORC1 signaling. Rather, Rheb-mediated synapse overgrowth was rescued by knockout of either of two TORC2 components, *rictor* or *sin1*. *Sin1* mutants also displayed a smaller synapse compare to controls, indicating that TORC2 serves a role in normal synapse growth. A recent study also discovered a role for TorC2 in the growth of sensory neuron dendritic arbors in *Drosophila* [9], thus TorC2 is clearly an important element in Tor regulation of neuron and synapse morphogenesis.

The inability of *raptor or S6k* RNAi to suppress Rheb-directed synapse overgrowth, together with the clear effects of *rictor* and *sin1* on this phenotype, emphasizes that hyperactivation of the Tor pathway in neurons produces NMJ expansion at least in some measure via a TorC2-directed process. Paradoxically, however, knockdown of the downstream TorC1 effector *S6k* produced a significant increase in the severity of this *Rheb*-mediated synapse overgrowth phenotype. In situations where Tor signaling is elevated, such as occurs when *Rheb* is overexpressed, an inhibitory feedback mechanism exists between S6k and the insulin receptor substrate Chico that dampens the level of Tor pathway activation [45]. Knockdown of S6k in the context of *Rheb* overexpression decreases or eliminates this feedback and could result in even higher levels of Tor activation. It is possible that this loss of S6k feedback indirectly increases the level of TorC2 activation, resulting in the enhancement of synapse overgrowth we see in these animals.

Although it is widely accepted that TorC1 is directly activated by Rheb [20], [34], the relationship between Rheb and TorC2 is not fully understood. Studies using cultured *Drosophila* S2 cells previously suggested an inhibitory effect of Rheb on TorC2 activity [16], [46], yet this model doesn't fit with our observations *in vivo*. Overexpression of *Rheb* caused synapse overgrowth at the NMJ which could be rescued by knockout of TorC2 components *rictor* and *sin1*. Rather than an inhibitory relationship, this finding suggests a positive relationship between Rheb and TorC2 in this context. Whether Rheb can directly activate TorC2 or must activate it through some indirect process remains to be explored, but the basic relationship is clear. The observation that Tor pathway components can behave differently in various cellular contexts is not without precedent. In the mouse brain, for example, inactivation of *PDK1*, a critical mediator of PI3K signaling, caused an increase in phosphorylated AKT at the TORC2-dependent site, but this effect was seen only in glial cells and not in neurons [47]. Likewise, *AMPK* mutations have been shown to cause cell-polarity defects in *Drosophila* epithelial tissues [21], yet in the retinal epithelium *AMPK* mutations caused a progressive neural degeneration phenotype and cell polarity was normal [48]. Even within cells of the same type there is evidence that Tor-signaling components interact with each other differently based on changes in the cellular environment. In the *Drosophila* wing disk, for example, under normal physiological conditions feedback inhibition of Akt phosphorylation is primarily mediated through an S6k-independent function of TorC1, yet under conditions of elevated Tor pathway activity, feedback inhibition becomes S6k-dependent [45]. Taken as a whole, these observations show that the relationships between different Tor-signaling components are cell-type specific and respond dynamically to different signaling states.

Recently autophagy has been shown to affect NMJ expansion via the regulation of Highwire (Hiw), a ubiquitin E3 ligase [49]. Activation of autophagy, either by reduction of TorC1 signaling or overexpression of *atg1*, produced a characteristic synapse expansion phenotype with long synaptic branches and many small diameter boutons. Loss of *hiw*, in addition to producing a larger NMJ, compromises the physiological function of the NMJ and results in markedly reduced EJP responses [50], [51]. We consider this type of synapse expansion to be functionally distinct from what we have produced in our *Drosophila* model of TS, where hyperactivation of Tor signaling produces a large synapse with many large boutons and an enhanced EJP response to suprathreshold stimulation of the motoneuron. The involvement of TorC1-directed autophagy in regulating the levels of Hiw, a modulator of synapse growth, and the clear role of TorC2 in the synapse expansion we describe here, emphasizes the complexity of Tor function in synapse development.

### Implications from a fly model of TS

Understanding the complex relationships between various inputs to Tor is important for designing interventions that could ameliorate the neurological and behavioral consequences of elevated TOR signaling, as occurs in humans with TS. In traditional metabolic disorders such as phenylketonuria, nervous system function is disrupted by a buildup of toxic intermediaries produced by deficits in particular metabolic pathways [52]. Disruptions of TOR signaling operate much differently, where it is the levels of common metabolic regulatory molecules that must be tightly controlled to ensure appropriate activation of the pathway. Our results using this relatively simple model system suggest that pharmaceutical interventions at different levels of TOR signaling may have very different effects with respect to neurological function. Neurological deficits of TS individuals might best be affected by Rheb-directed interventions, since that level of the pathway affects both axon guidance and synapse assembly. Targeting more distant inputs must be done carefully, since some of these may have unanticipated effects. For example, AMPK expression substantially diminished axon guidance and behavioral deficits, but it also caused a marked increase in the severity of synaptic overgrowth and hyperfunctionality. Similarly, although TORC1-specific drugs such as rapamycin can be used to treat the effects of TOR-hyperactivation in humans, these will likely not affect TORC2-mediated abnormalities which could contribute to some elements of the neurological or behavioral deficits. The possibility of ameliorating TOR-misregulation with diet suggests a viable alternative to pharmaceutical intervention. Diet restriction has long been used to effectively treat seizure disorders [24], but a better understanding of this pathway is needed to make optimal decisions about the dietary changes that would be most effective and clinically realistic. Clearly, the more we come to understand about this unique system that links metabolism and neural development, the better equipped we will be to address the various problems that arise when control of this pathway is lost.

## Materials and Methods

### Fly Stocks

w^*^; P{UAS-Rheb.Pa}3 (*UAS-Rheb^+^*), w^*^; P[w^+^;Gal4-elav.L]2/CyO (*elav-Gal4*), *P{GawB}elav^C155^* (*elav^C155^*), *Oregon-R-C* (*Oregon-R*), y^1^ w^*^; P{WT-Dp110}2 (*UAS-Pi3K^WT^*), *P{Dp110-CAAX}1* (*UAS-Pi3K^CAAX^*), and *w^1118^; PBac{RB}Sin1^e03756^* (*Sin1^−/−^*) were from the Bloomington *Drosophila* Stock Center. *P{GD5138}v13112/CyO* (*UAS-raptor^RNAi^*) and *w^1118^ P{GD6646}v18126* (*UAS-S6K^RNAi^*) were from the Vienna *Drosophila* RNAi Center. *Tor^Δ6B^* was a gift from T. Neufeld, *UAS-AMPK^TD^ 4-1* was a gift from J. Chung [21], and both *rictor^Δ1^* and *rictor^Δ2^* were gifts from S. Cohen [13].

### Food Preparation

Our standard fly food contains approximately 869 kcal/L from the cornmeal, sugar and yeast ingredients (Harvard Biolabs). The four diets were taken directly from a recipe previously described [36]. Per Liter of food, all diets contained 20 g of agar, 30 mL of a 100 g/L methyl 4-hydroxybenzoate (Sigma) solution, 3 mL of propionic acid, and varying amounts of brewer's yeast and sucrose depending on the diet. The rich, high-calorie (HC) diet contained 150 g/L of both yeast and sucrose resulting in an estimated total of 1203 kcal/L, the yeast-restricted (YR) diet contained 65 g/L of yeast and 150 g/L of sucrose for an estimated total of 861 kcal/L, the sugar-restricted (SR) diet contained 150 g/L of yeast and 65 g/L of sucrose for an estimated total of 863 kcal/L, and the calorie-restricted (CR) diet contained 65 g/L of both yeast and sucrose for an estimated total of 521 kcal/L.

### Phototaxis analysis

Phototaxis analysis was performed using an 11-tube countercurrent distribution apparatus similar to one previously described [53] and based on the phototaxis sorting technique developed by S. Benzer [54]. Newly-eclosed *Drosophila* adults were sedated by carbon dioxide and counted into sample groups of 50–100 mixed male and female populations. After a 24-hour period of recovery, the flies were dark-adapted (placed in a dark room) for 30 minutes and then transferred into the first tube of the phototaxis apparatus. A 15-watt soft-white fluorescent lamp was placed 6 cm away, and the flies were allowed 60 seconds between each shift of the apparatus to move towards the lighted end of each tube. After ten trials, the number of flies in each tube was counted and the results were then analyzed using the phototaxis index equation, 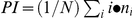 where *i* is the fraction number (tube number), *n* is the number of flies in the given fraction, and *N* is the total number of flies tested [31].

### Immunohistochemistry

For visualization of photoreceptors, *Drosophila* were dissected 40-hours after pupal formation and stained according to established protocols [55]. Anti-Chaoptin (MAb 24B10, Developmental Studies Hybridoma Bank) was used at 1∶25. The widths of misrouted axon bundles were measured using ImageJ data analysis software (NIH). For visualization of NMJ synapses, third instar larvae were filleted in PBS and fixed in 4% formaldehyde before staining with anti-Cysteine String Protein at 1∶1000 (MAb 1G12, Developmental Studies Hybridoma Bank). For determining “bouton pixel number,” a measure of synapse size, individual confocal optical sections were volume-rendered to show the entire depth of Anti-CSP staining for each synapse (between muscles 6 and 7 of the second abdominal (A2) hemisegment). Traditionally, synapse size at the NMJ has been assessed by counting boutons detected by anti-CSP or other presynaptic marker staining in a two dimensional projection of a serially-sectioned preparation. We found this method limiting in conditions where individual boutons are not distinct and it is difficult to obtain a reliable bouton count. We therefore measured synapse size by determining the number of anti-CSP stained pixels, setting a threshold for staining above background. Images were opened in Adobe Photoshop CS3 (Adobe Systems Inc., San Jose, CA) and a threshold value was set for each image to maximize the synapse while removing background fluorescence. Pixels below threshold were assigned an intensity value of 0 and all pixels above threshold intensity were assigned a value of 255. A histogram function was used to measure the total number of pixels within the synapse that were above threshold. This method of synapse size measurement provides for quantitation where the subjective assignment of whether fluorescence is coalesced into a defined bouton is not part of the process. This method was compared to the standard bouton-counting procedure, where individual boutons are scored, and similar relationships between experimental samples were obtained. For example, the bouton-counting method (normalized for muscle area) for *elav-Gal4>UAS-Rheb* animals reared on different diets gave the following measures (HC = 0.0029±0.000188, YR = 0.00275±0.00018, SR = 0.00335±0.000164 and CR = 0.00283±0.00020 with n = 30, 25, 32 and 12 respectively). Note that the relationships between the different data sets are the same as when assessed using the pixel counting method (shown in Figure 6D), with one sample significantly different (larger) than the others (SR diet). In all cases, muscle surface areas were the combined measurements of muscles 6 and 7 as determined by ImageJ data analysis software (NIH,Bethesda, MD). Images were collected using a Nikon C1 upright laser confocal and Olympus FV1000 laser scanning confocal microscope with Nikon EZC1 imaging software and Imaris V7.3 (Bitplane Inc. Saint Paul, MN).

### Electrophysiology

EJP recordings were taken from muscle six of the second abdominal hemisegment (A2) in third instar larvae. Dissections were performed in calcium-free saline and recordings were taken in modified HL3 medium with a calcium concentration of 1.2 mM [39]. Thin-walled glass recording electrodes with resistances of 10–30 mΩ were pulled on a Model P-87 needle puller (Sutter Instrument Company) and back-filled with 3 M KCl. Muscle six of A2 was impaled with the recording electrode, and a suction electrode was used to stimulate the motoneuron with a Grass stimulator delivering six 1 ms pulses at a frequency of 0.1 Hz and an intensity of approximately 1.5 times the minimum required to evoke a compound response. Recordings were acquired with an Axoclamp 2B amplifier and Clampex 9.2 software (Axon Instruments). EJP amplitudes were measured with MiniAnalysis software from Synaptosoft.

### Food Consumption Analysis

Rates of ingestion and nutrient absorption were measured based on previously published techniques [38]. Flies were reared on four different diets from hatching to early third instar, at which point they were transferred to new vials containing the same food seeded with 10 µCi/mL of dCTP[α-32P] (MP Biomedicals). After six hours of free feeding, 10–15 larvae from each group were washed and placed in 10 mL of scintillation fluid. Accumulated levels of radioactivity were then measured using a Beckman Liquid Scintillation counter and the amount of ingested food was calculated.

## Supporting Information

Figure S1 **Diets that vary in nutritional content are consumed and absorbed equally by third instar larvae.** Food ingestion was measured for *elav-Gal4>UAS-Rheb^+^* third instar larvae raised on four different diets. Despite having varying nutritional content, each of the foods we tested was consumed approximately equally during a six hour period of free feeding. Although there appeared to be a trend towards increased feeding on the SR diet, the difference was not statistically significant (p≤0.39 in a two-tailed Student's t-test compared against HC food).
(EPS)
